# Wholer’s Outlines of Organic Chemistry

**Published:** 1873-02

**Authors:** 


### Books Reviewed.

---

*Wholer's Outlines of Organic Chemistry.* By Rudolph Fittig, Ph. D., Nat. Sc. D. Translated from the eighth German edition, with editions. By Ira Remsen, M. D., Ph. D. Philadelphia: Henry C. Lea, 1873. Buffalo: T. Butler & Son.

The work before us has reached a deservedly high reputation in Germany, and is placed before the American reader with the idea that it will be of value both to the beginner and the advanced student, and that it will serve as a valuable text book to all students of organic chemistry. A rapid review of the work serves to confirm this opinion and induces us to recommend the book as a work worthy of careful study. The editor of the American edition has introduced a chapter on the "Constitution of Chemical Compounds," to assist the beginner to comprehend the terms which might otherwise puzzle him.

To make the work of more value to medical students a section on Animal Chemistry is introduced, which, for the small amount of space necessarily allotted it, is very comprehensive.

We have been much pleased at the simplicity and conciseness displayed, and are happy to welcome it as another addition to the literature of the profession.

